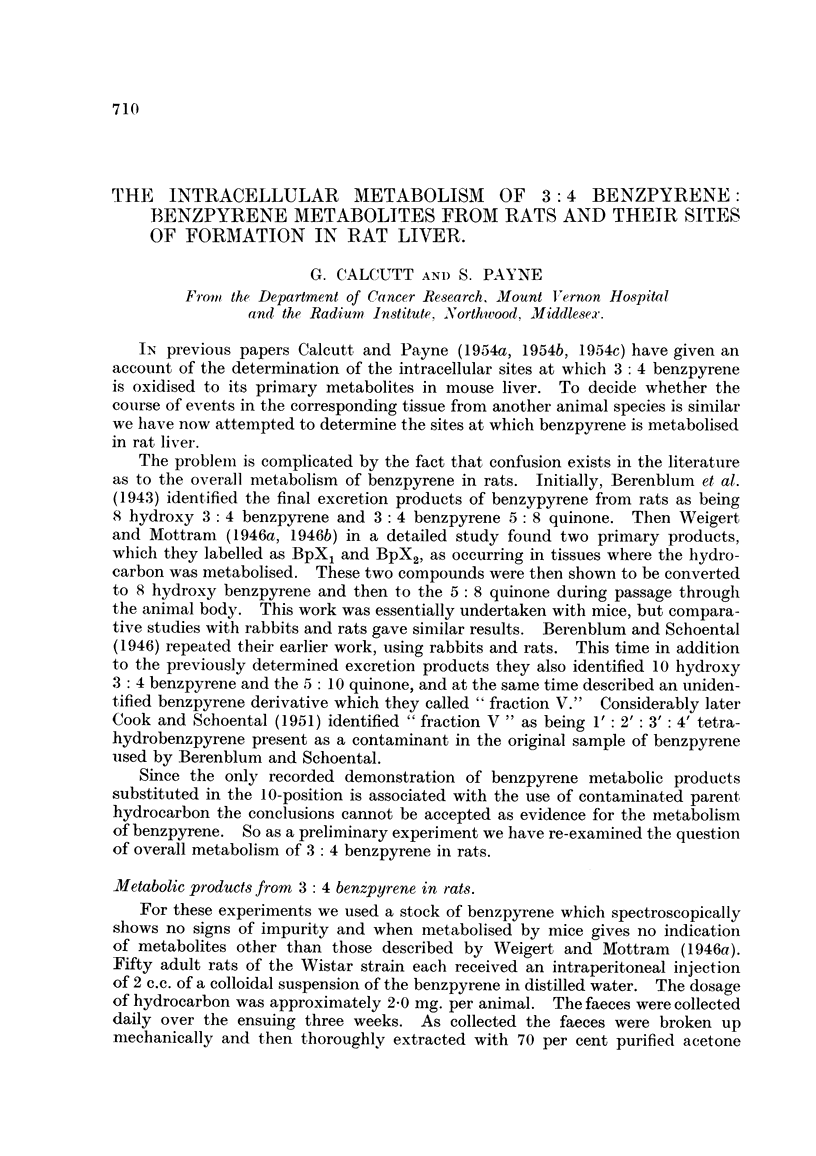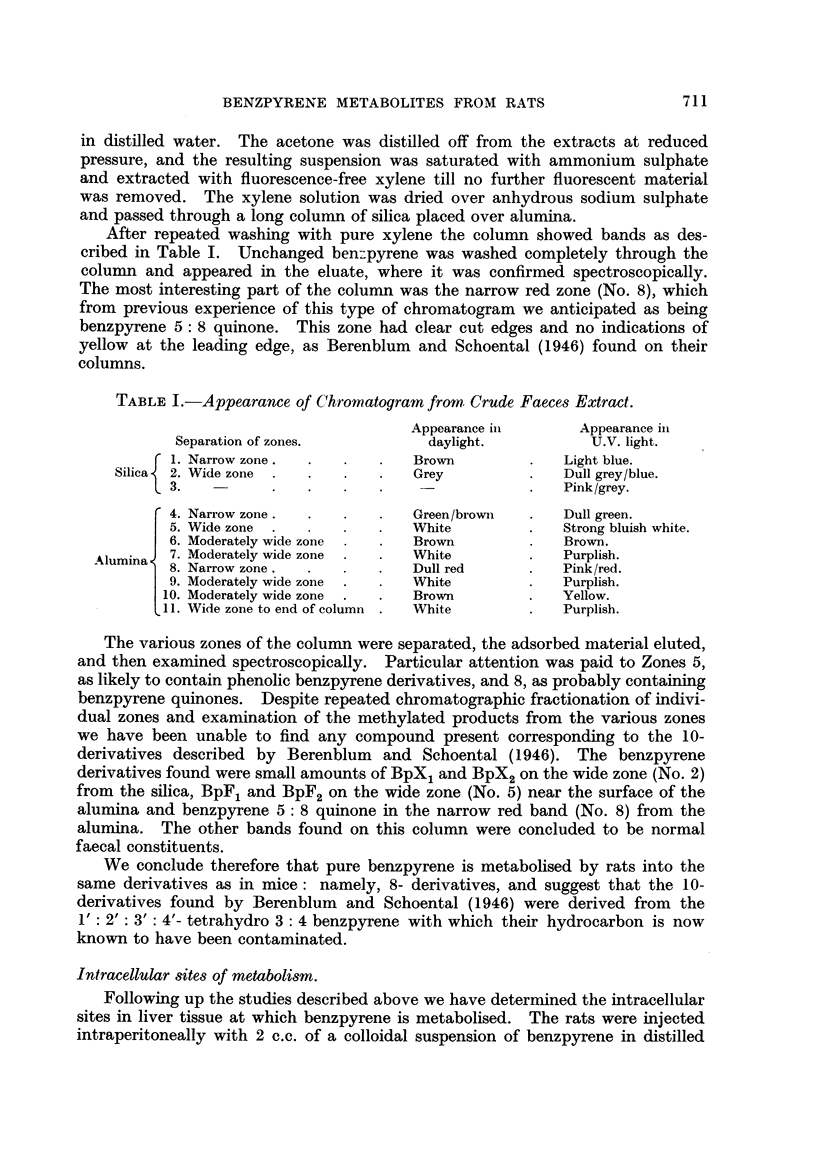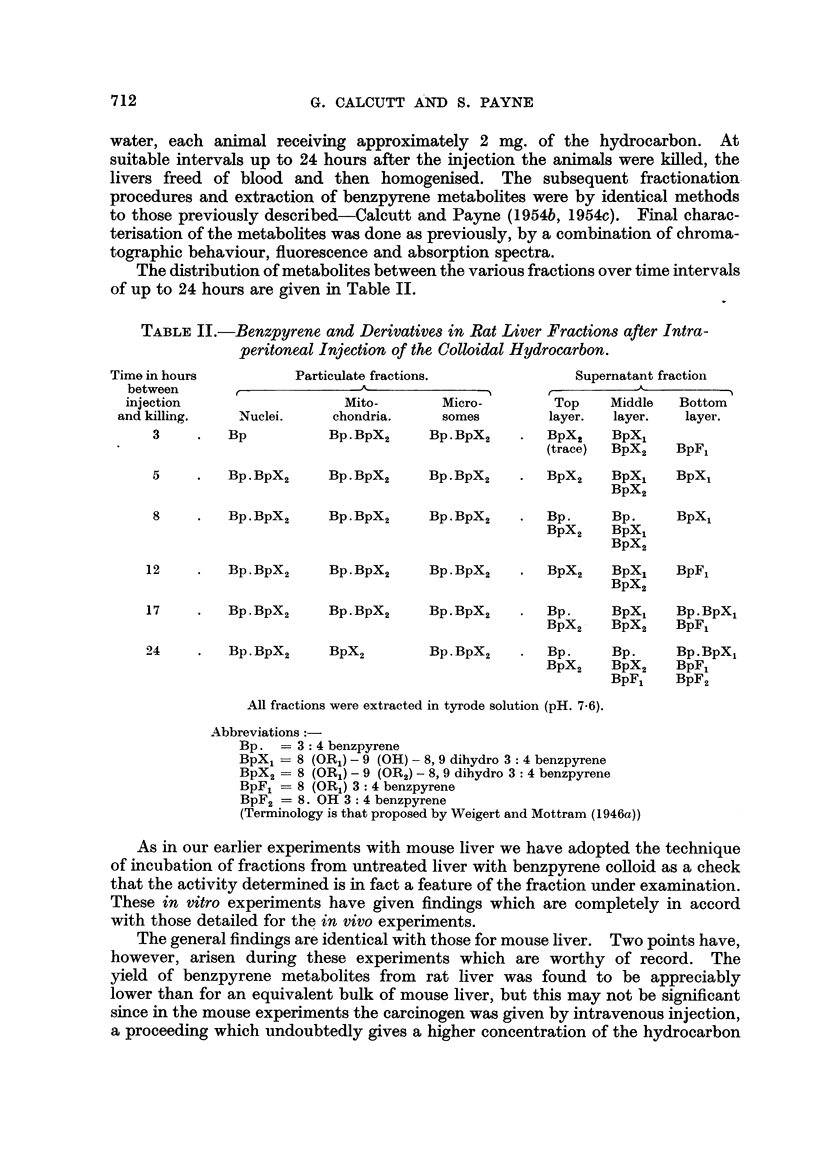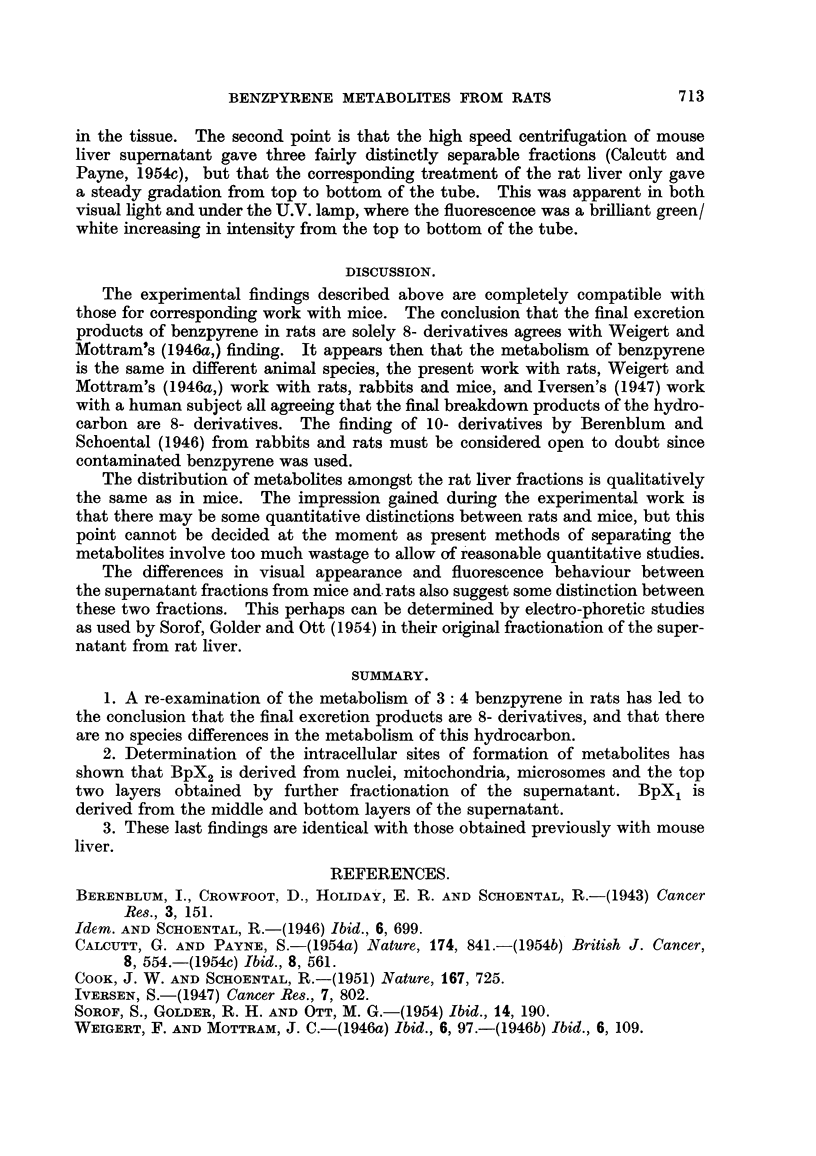# The Intracellular Metabolism of 3: 4 Benzpyrene: Benzpyrene Metabolites from Rats and their Sites of Formation in Rat Liver

**DOI:** 10.1038/bjc.1954.78

**Published:** 1954-12

**Authors:** G. Calcutt, S. Payne


### THE INTRACELLULAR METABOLISM OF 3 : 4 BENZPYRENE: BENZPYRENE METABOLITES FROM RATS AND THEIR SITES OF FORMATION IN RAT LIVER.

G. CALCUTT AND S. PAYNE

*From the Department of Cancer Research, Mount Vernon Hospital and the Radium Institute, Northwood, Middlesex.*

IN previous papers Calcutt and Payne (1954*a*, 1954*b*, 1954*c*) have given an account of the determination of the intracellular sites at which 3 : 4 benzpyrene is oxidised to its primary metabolites in mouse liver. To decide whether the course of events in the corresponding tissue from another animal species is similar we have now attempted to determine the sites at which benzpyrene is metabolised in rat liver.

The problem is complicated by the fact that confusion exists in the literature as to the overall metabolism of benzpyrene in rats. Initially, Berenblum *et al.* (1943) identified the final excretion products of benzypyrene from rats as being 8 hydroxy 3 : 4 benzpyrene and 3 : 4 benzpyrene 5 : 8 quinone. Then Weigert and Mottram (1946*a*, 1946*b*) in a detailed study found two primary products, which they labelled as $BpX_1$ and $BpX_2$, as occurring in tissues where the hydrocarbon was metabolised. These two compounds were then shown to be converted to 8 hydroxy benzpyrene and then to the 5 : 8 quinone during passage through the animal body. This work was essentially undertaken with mice, but comparative studies with rabbits and rats gave similar results. Berenblum and Schoental (1946) repeated their earlier work, using rabbits and rats. This time in addition to the previously determined excretion products they also identified 10 hydroxy 3 : 4 benzpyrene and the 5 : 10 quinone, and at the same time described an unidentified benzpyrene derivative which they called " fraction V." Considerably later Cook and Schoental (1951) identified " fraction V " as being 1′ : 2′ : 3′ : 4′ tetrahydrobenzpyrene present as a contaminant in the original sample of benzpyrene used by Berenblum and Schoental.

Since the only recorded demonstration of benzpyrene metabolic products substituted in the 10-position is associated with the use of contaminated parent hydrocarbon the conclusions cannot be accepted as evidence for the metabolism of benzpyrene. So as a preliminary experiment we have re-examined the question of overall metabolism of 3 : 4 benzpyrene in rats.

*Metabolic products from 3 : 4 benzpyrene in rats.*

For these experiments we used a stock of benzpyrene which spectroscopically shows no signs of impurity and when metabolised by mice gives no indication of metabolites other than those described by Weigert and Mottram (1946*a*). Fifty adult rats of the Wistar strain each received an intraperitoneal injection of 2 c.c. of a colloidal suspension of the benzpyrene in distilled water. The dosage of hydrocarbon was approximately 2·0 mg. per animal. The faeces were collected daily over the ensuing three weeks. As collected the faeces were broken up mechanically and then thoroughly extracted with 70 per cent purified acetone in distilled water. The acetone was distilled off from the extracts at reduced pressure, and the resulting suspension was saturated with ammonium sulphate and extracted with fluorescence-free xylene till no further fluorescent material was removed. The xylene solution was dried over anhydrous sodium sulphate and passed through a long column of silica placed over alumina.

After repeated washing with pure xylene the column showed bands as described in Table I. Unchanged benzpyrene was washed completely through the column and appeared in the eluate, where it was confirmed spectroscopically. The most interesting part of the column was the narrow red zone (No. 8), which from previous experience of this type of chromatogram we anticipated as being benzpyrene 5 : 8 quinone. This zone had clear cut edges and no indications of yellow at the leading edge, as Berenblum and Schoental (1946) found on their columns.

TABLE I.—*Appearance of Chromatogram from Crude Faeces Extract.*

| | Separation of zones. | Appearance in daylight. | Appearance in U.V. light. |
|---|---|---|---|
| Silica | 1. Narrow zone | Brown | Light blue. |
| | 2. Wide zone | Grey | Dull grey/blue. |
| | 3. — | — | Pink/grey. |
| Alumina | 4. Narrow zone | Green/brown | Dull green. |
| | 5. Wide zone | White | Strong bluish white. |
| | 6. Moderately wide zone | Brown | Brown. |
| | 7. Moderately wide zone | White | Purplish. |
| | 8. Narrow zone | Dull red | Pink/red. |
| | 9. Moderately wide zone | White | Purplish. |
| | 10. Moderately wide zone | Brown | Yellow. |
| | 11. Wide zone to end of column | White | Purplish. |

The various zones of the column were separated, the adsorbed material eluted, and then examined spectroscopically. Particular attention was paid to Zones 5, as likely to contain phenolic benzpyrene derivatives, and 8, as probably containing benzpyrene quinones. Despite repeated chromatographic fractionation of individual zones and examination of the methylated products from the various zones we have been unable to find any compound present corresponding to the 10-derivatives described by Berenblum and Schoental (1946). The benzpyrene derivatives found were small amounts of $BpX_1$ and $BpX_2$ on the wide zone (No. 2) from the silica, $BpF_1$ and $BpF_2$ on the wide zone (No. 5) near the surface of the alumina and benzpyrene 5 : 8 quinone in the narrow red band (No. 8) from the alumina. The other bands found on this column were concluded to be normal faecal constituents.

We conclude therefore that pure benzpyrene is metabolised by rats into the same derivatives as in mice : namely, 8- derivatives, and suggest that the 10-derivatives found by Berenblum and Schoental (1946) were derived from the 1′ : 2′ : 3′ : 4′- tetrahydro 3 : 4 benzpyrene with which their hydrocarbon is now known to have been contaminated.

*Intracellular sites of metabolism.*

Following up the studies described above we have determined the intracellular sites in liver tissue at which benzpyrene is metabolised. The rats were injected intraperitoneally with 2 c.c. of a colloidal suspension of benzpyrene in distilled water, each animal receiving approximately 2 mg. of the hydrocarbon. At suitable intervals up to 24 hours after the injection the animals were killed, the livers freed of blood and then homogenised. The subsequent fractionation procedures and extraction of benzpyrene metabolites were by identical methods to those previously described—Calcutt and Payne (1954*b*, 1954*c*). Final characterisation of the metabolites was done as previously, by a combination of chromatographic behaviour, fluorescence and absorption spectra.

The distribution of metabolites between the various fractions over time intervals of up to 24 hours are given in Table II.

TABLE II.—*Benzpyrene and Derivatives in Rat Liver Fractions after Intraperitoneal Injection of the Colloidal Hydrocarbon.*

| Time in hours between injection and killing. | Particulate fractions. | | | Supernatant fraction | | |
|---|---|---|---|---|---|---|
| | Nuclei. | Mito-chondria. | Micro-somes | Top layer. | Middle layer. | Bottom layer. |
| 3 | Bp | Bp.$BpX_2$ | Bp.$BpX_2$ | $BpX_2$<br>(trace) | $BpX_1$<br>$BpX_2$ | $BpF_1$ |
| 5 | Bp.$BpX_2$ | Bp.$BpX_2$ | Bp.$BpX_2$ | $BpX_2$ | $BpX_1$<br>$BpX_2$ | $BpX_1$ |
| 8 | Bp.$BpX_2$ | Bp.$BpX_2$ | Bp.$BpX_2$ | Bp.<br>$BpX_2$ | Bp.<br>$BpX_1$<br>$BpX_2$ | $BpX_1$ |
| 12 | Bp.$BpX_2$ | Bp.$BpX_2$ | Bp.$BpX_2$ | $BpX_2$ | $BpX_1$<br>$BpX_2$ | $BpF_1$ |
| 17 | Bp.$BpX_2$ | Bp.$BpX_2$ | Bp.$BpX_2$ | Bp.<br>$BpX_2$ | $BpX_1$<br>$BpX_2$ | Bp.$BpX_1$<br>$BpF_1$ |
| 24 | Bp.$BpX_2$ | $BpX_2$ | Bp.$BpX_2$ | Bp.<br>$BpX_2$ | Bp.<br>$BpX_2$<br>$BpF_1$ | Bp.$BpX_1$<br>$BpF_1$<br>$BpF_2$ |

All fractions were extracted in tyrode solution (pH. 7·6).

Abbreviations :—
Bp. = 3 : 4 benzpyrene
$BpX_1$ = 8 $(OR_1)$ – 9 (OH) – 8, 9 dihydro 3 : 4 benzpyrene
$BpX_2$ = 8 $(OR_1)$ – 9 $(OR_2)$ – 8, 9 dihydro 3 : 4 benzpyrene
$BpF_1$ = 8 $(OR_1)$ 3 : 4 benzpyrene
$BpF_2$ = 8. OH 3 : 4 benzpyrene
(Terminology is that proposed by Weigert and Mottram (1946*a*))

As in our earlier experiments with mouse liver we have adopted the technique of incubation of fractions from untreated liver with benzpyrene colloid as a check that the activity determined is in fact a feature of the fraction under examination. These *in vitro* experiments have given findings which are completely in accord with those detailed for the *in vivo* experiments.

The general findings are identical with those for mouse liver. Two points have, however, arisen during these experiments which are worthy of record. The yield of benzpyrene metabolites from rat liver was found to be appreciably lower than for an equivalent bulk of mouse liver, but this may not be significant since in the mouse experiments the carcinogen was given by intravenous injection, a proceeding which undoubtedly gives a higher concentration of the hydrocarbon in the tissue. The second point is that the high speed centrifugation of mouse liver supernatant gave three fairly distinctly separable fractions (Calcutt and Payne, 1954*c*), but that the corresponding treatment of the rat liver only gave a steady gradation from top to bottom of the tube. This was apparent in both visual light and under the U.V. lamp, where the fluorescence was a brilliant green/white increasing in intensity from the top to bottom of the tube.

### DISCUSSION.

The experimental findings described above are completely compatible with those for corresponding work with mice. The conclusion that the final excretion products of benzpyrene in rats are solely 8- derivatives agrees with Weigert and Mottram's (1946*a*,) finding. It appears then that the metabolism of benzpyrene is the same in different animal species, the present work with rats, Weigert and Mottram's (1946*a*,) work with rats, rabbits and mice, and Iversen's (1947) work with a human subject all agreeing that the final breakdown products of the hydrocarbon are 8- derivatives. The finding of 10- derivatives by Berenblum and Schoental (1946) from rabbits and rats must be considered open to doubt since contaminated benzpyrene was used.

The distribution of metabolites amongst the rat liver fractions is qualitatively the same as in mice. The impression gained during the experimental work is that there may be some quantitative distinctions between rats and mice, but this point cannot be decided at the moment as present methods of separating the metabolites involve too much wastage to allow of reasonable quantitative studies.

The differences in visual appearance and fluorescence behaviour between the supernatant fractions from mice and rats also suggest some distinction between these two fractions. This perhaps can be determined by electro-phoretic studies as used by Sorof, Golder and Ott (1954) in their original fractionation of the supernatant from rat liver.

### SUMMARY.

1. A re-examination of the metabolism of 3 : 4 benzpyrene in rats has led to the conclusion that the final excretion products are 8- derivatives, and that there are no species differences in the metabolism of this hydrocarbon.

2. Determination of the intracellular sites of formation of metabolites has shown that $BpX_2$ is derived from nuclei, mitochondria, microsomes and the top two layers obtained by further fractionation of the supernatant. $BpX_1$ is derived from the middle and bottom layers of the supernatant.

3. These last findings are identical with those obtained previously with mouse liver.

### REFERENCES.

BERENBLUM, I., CROWFOOT, D., HOLIDAY, E. R. AND SCHOENTAL, R.—(1943) *Cancer Res.*, **3**, 151.

*Idem.* AND SCHOENTAL, R.—(1946) *Ibid.*, **6**, 699.

CALCUTT, G. AND PAYNE, S.—(1954*a*) *Nature*, **174**, 841.—(1954*b*) *British J. Cancer*, **8**, 554.—(1954*c*) *Ibid.*, **8**, 561.

COOK, J. W. AND SCHOENTAL, R.—(1951) *Nature*, **167**, 725.

IVERSEN, S.—(1947) *Cancer Res.*, **7**, 802.

SOROF, S., GOLDER, R. H. AND OTT, M. G.—(1954) *Ibid.*, **14**, 190.

WEIGERT, F. AND MOTTRAM, J. C.—(1946*a*) *Ibid.*, **6**, 97.—(1946*b*) *Ibid.*, **6**, 109.